# Scrotal Flaps for Penile Skin Reconstruction: A Systematic Review

**DOI:** 10.3390/medicina61061052

**Published:** 2025-06-06

**Authors:** Sorin V. Parasca, Andrei Dumitrescu, Florin R. Stanescu, Ruxandra D. Sinescu

**Affiliations:** 1 Faculty of Medicine, Carol Davila University of Medicine and Pharmacy, 020021 Bucharest, Romania; sorin.parasca@umfcd.ro (S.V.P.); ruxandra.sinescu@umfcd.ro (R.D.S.); 2 Emergency Hospital for Plastic Surgery and Burns, 218 Calea Grivitei, Sector 1, 010761 Bucharest, Romania; florin-radu.stanescu@rez.umfcd.ro; 3 Elias Emergency University Hospital, 011461 Bucharest, Romania

**Keywords:** scrotal flaps, penile resurfacing, sclerosing lipogranuloma, penile reconstruction

## Abstract

*Background and Objectives*: Infection, trauma, skin cancer, foreign substance injections and lymphedema are among the most frequent causes of penile skin defects. Scrotal flaps are a promising reconstructive option for penile resurfacing, offering improved functional and aesthetic outcomes; however, there is no clear consensus on their superiority. *Materials and Methods*: A review of the literature was performed in PubMed Central and Scopus, and multiple keywords were employed. The initial search retrieved 9181 articles; 32 articles were finally selected, of which 13 were case reports and 19 were case series. *Results*: A total of 368 patients were included, the majority (71%) consulting for sclerosing lipogranuloma. Seven types of scrotal flaps were used: unilateral scrotal flap (n = 1), bilateral anterior scrotal flaps (n = 149), two-stage scrotal flap (n = 57), bipedicled bilateral anterior scrotal flaps (n = 140), apron-style scrotal flap (n = 1), scrotal pull-up (n = 13), and island dartos musculocutaneous flap (n = 7). Patient satisfaction was high in all studies. Outcome evaluation was typically conducted using subjective questionnaires with 2 or 5 items or visual analog scales. Few studies employed validated sexual function questionnaires, as the IIEF-5 or the EHS. *Conclusions*: Scrotal flaps provide good quality tissue for penile resurfacing, having the closest resemblance to normal penis skin. For a better understanding of the outcomes of different scrotal flaps, a thorough evaluation of postoperative complications should be made. The LOS and revision surgery rates may serve as surrogates for the financial burden of the procedure. Erectile function should be thoroughly evaluated with a 10-item Likert scale, IIEF-5, EHS, and POSAS.

## 1. Introduction

Penile sclerosing lipogranuloma, also known as siliconoma, oleogranuloma, and vaselinoma, is an entity caused by injecting lipid-rich foreign substances in the penis and/or scrotum for aesthetic purposes (girth increase) [1,2,3]. The latency of complication onset is variable, ranging from 1 day to more than 20 years [4]. Complications following injection include of penile skin necrosis, foreign body granuloma formation, phimosis, erectile disfunction, and voiding difficulties, and treatment almost always generates a circumferential full-thickness skin defect [5,6,7,8].

Penile trauma including avulsion injuries, bites, burns, and iatrogenic injury from excessive electric cautery use during circumcision can lead to partial or circumferential penile skin defects. This tissue loss may result from the initial causative agent or be a consequence of surgery [9,10,11,12,13,14,15].

Buried penis syndrome, also known as concealed penis, is a rare condition, affecting both children and adults. The penile shaft is of normal length but is covered by excess peripenile tissue, reducing its external exposure and often making sexual intercourse difficult or impossible [16]. Surgery is recommended when it does not spontaneously resolve [17].

Skin grafting is the most common reconstructive option for circumferential penile shaft skin loss given its easy, fast application; however, it often yields suboptimal results [18,19]. For more complex defects, local, regional, and free flaps have been extensively used to provide soft tissue coverage for the genital area but bulkiness, abnormal appearance, and lack of sensibility remain problems that must be addressed to achieve a satisfactory result [2,3,4,20,21,22].

Scrotal flaps seem to be a better reconstructive option for penile shaft defects due to their similar skin quality, sensibility, and elasticity. They provide a “more natural” appearance and improved functional and aesthetic outcomes [1,12,14,15,19,23].

The main objective of this review is to analyze which type of scrotal flaps employed for circumferential penile skin defect reconstruction in the adult population have the best clinical outcome. The secondary objectives were to assess the types of complications associated with different flap techniques, to evaluate what flap provides the best patient-reported satisfaction score, to review the reporting standards in the current literature, and to identify knowledge gaps for future research on this topic.

## 2. Materials and Methods

This systematic review was conducted using the Preferred Reporting Items for Systematic Reviews and Meta-Analysis (PRISMA) framework as a general guide for the study selection process, and the PRISMA flowchart was included to illustrate the article selection sequence. The PRISMA checklist was consulted to inform the structure of the review, but not all elements were applied due to the specific aims and scope of this study [24,25]. A search of PubMed and Scopus databases was performed, employing the following search string (“scrotal, flaps” [MeSH Terms] OR “scrotal flaps” OR “dartos myofasciocutaneous flap” OR “dartos muculocutaneous flap” OR “paraffinoma” OR “lipogranuloma” OR “penile reconstruction” OR “penis resurfacing” OR “penile skin defect” OR “penile siliconoma”). Articles were included from inception to the search date, with inclusion criteria encompassing case reports, case series, reviews and full-text articles written in English that focused on human cases in the adult population and having a description of the specific scrotal flap technique used. Studies involving the use of scrotal flaps in pediatric patients or for hypospadias repair were excluded.

**Figure 1:**
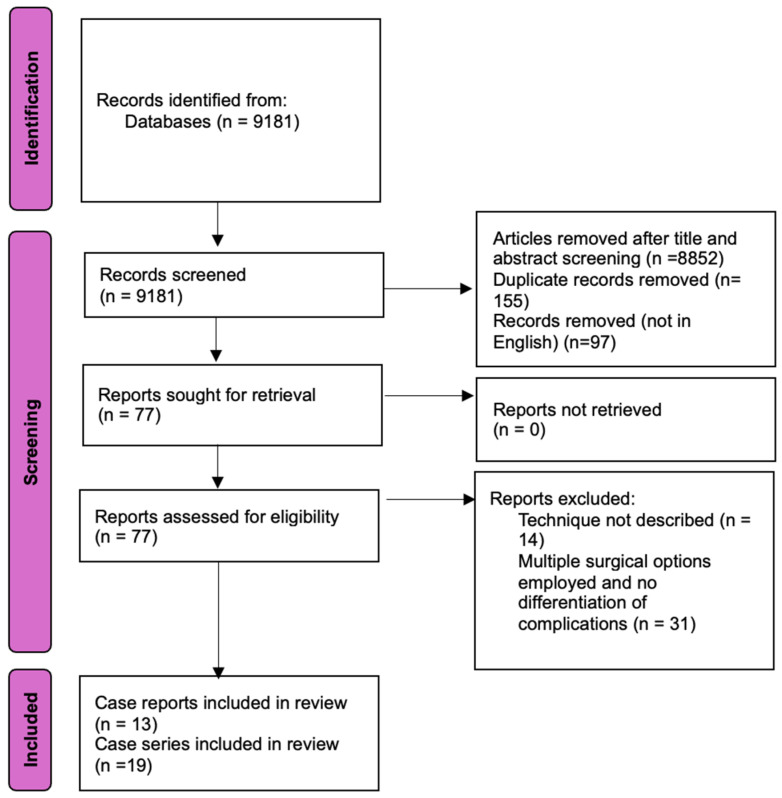
PRISMA chart showing article selection sequence.

The initial search retrieved 9181 articles. After the screening for titles and abstracts, 329 articles were evaluated. Some 97 articles were excluded because they were not written in English, and 155 were excluded for being duplicates. Further selection for eligibility was conducted, which led to the exclusion of 45 papers. A total of 32 articles were finally selected for analysis, of which 13 were case reports and 19 were case series (Figure 1). Paper publication dates ranged from 1991 to 2024.

Data collection included first author, year of publication, mean age of the patients, cause of skin defect, presenting symptoms, type of skin defect, anatomical level of debridement, surgical technique employed, number of operative stages, type of anesthesia, operative time, length of hospital stay, complications (dehiscence, partial/total necrosis, hematoma, infections, scar contracture, sexual function disorder, urethral fistula), revision surgery, time to recovery, patient satisfaction data (questionnaires employed for sexual, aesthetic, erectile function evaluations), and mean follow-up. Risk of bias was assessed utilizing the Risk Of Bias In Non-randomized Studies–of Interventions (ROBINS-I). In the review process, two reviewers (A.D. and F.S.) independently screened all titles, abstracts, and full-text articles. Any disagreements were resolved through discussion with a third reviewer (S.P.) to ensure consensus.

## 3. Results

This study evaluated the reports of 368 patients with a mean age of 36.57 ± 9.21 (Table 1), the majority (71%) presenting for complications following foreign substance (paraffin, silicone, mineral oil) injections in the genital area and the rest consulted for complications following circumcision, buried penis, penile skin cancer, burns, animal bites, or trauma. The number of patients in each category is presented in Table 2.

The presenting symptoms in the sclerosing lipogranuloma group are synthesized in Table 3.

In five studies, the initial presentation symptoms were not specified [23,26,30,38,39].

Some 83% of the patients had circumferential defects requiring coverage, and debridement was carried out at Buck’s fascia level (not including it) in all cases, irrespective of the disease.

Seven types of scrotal flaps with a total of five variations were used in the analyzed data: unilateral scrotal flap (n = 1), bilateral anterior scrotal flaps with three variations (n = 149), two-stage scrotal flap with inlay (n = 57), bipedicled bilateral anterior scrotal flaps with two variations (n = 140), apron-style scrotal flap (n = 1), scrotal pull-up (n = 13). and island dartos musculocutaneous flap (n = 7).

The anterior bilateral scrotal flap was initially described by Jeong et al. as a new repair technique for penile paraffinoma [38]. They state the importance of preoperative assessment of the scrotal tissue and the need for proper stretch of the scrotal skin when designing the flap. The raphe incision is made first, and the flaps are elevated in the subdartos plane by blunt dissection and transposed to the penile shaft, suturing them to the prepuce or the glans. They also incorporated ventral and dorsal Z plasties to minimize scar contracture and the scrotum is closed in an inverted T fashion after the excision of lateral dog ears. Shin subsequently modified this technique by suturing the ventral skin in an inverted V, compared it to the traditional T-style repair, and stated that with this modification, fewer complications were encountered in their series regarding ventral skin necrosis or delayed wound healing; however, they did not provide statistical analysis of the data [30]. The method of Jeong was also used by Son for a patient with aquafilling-induced lipogranuloma. In addition, Son et al. reported doubling the vertical length of the flaps and deepithelializing half of it with subsequent infolding for girth auto-augmentation. They reported a very satisfactory outcome based on patient appreciation and suggested the utility of this technique to improve self-confidence [37].

The bilateral bipedicled scrotal flaps described by Muranyi et al. involve a horizontal incision in the scrotum through which a subdartos tunnel is created for denuded penis pull-through. This incision line is sutured to the subcoronal incision line, and the penoscrotal incision is closed primarily on the dorsal aspect of the penis. Next, a relaxing V incision is placed on the ventral aspect of the penis, which is subsequently closed in a linear pattern. They reported the theoretical advantage of reducing postoperative complications, as the suture lines are separated from one another. No T-anastomosis occurs, and the scrotal skin between the dorsal suture line and the subcoronal incision is in continuity transversally, which allows for tensionless erections [23].

Gao added a Z-plasty on the ventral aspect of the penis when using a bilateral bipedicled scrotal flap for penile coverage in a young man who suffered from skin necrosis and subsequent contracture after circumcision. They stated that in doing this, the ventral displacement of the penis after scar contracture is less likely to occur [9].

The apron-style flap uses the scrotal skin wrapped around the entire penile shaft, with no dorsal suture line, which generates a better cosmetic result. Nevertheless, there is a T-style anastomosis at the scrotal-subcoronal junction and two lateral suture lines at the penoscrotal junction that unite with the longitudinal ventral suture line which, at least in theory, augments the risk of delayed wound healing of marginal necrosis. That said, no complications were reported in the single case report included in this study [31].

The scrotal pull-up technique uses a rectangular, inferiorly based flap for coverage of ventral skin defects such as those encountered after concealed penis treatment and ventral penile neoplasia. The disadvantage of this method is that the closure is performed under tension, and the penoscrotal boundary is ill defined [11,17]. The advantage is that the procedure is easy to perform, fast, and does not require precise incorporation of scrotal artery branches.

The dartos musculocutaneous island flap is based on the anterior scrotal branches of the deep external pudendal arteries. This flap is useful for non-circumferential defects of moderate size or for patients with a small scrotum, reducing the iatrogenic scrotal contracture. Tiwari et al. reported six cases of island dartos myocutaneous flaps used to treat patients with postburn scars with good sensibility. They had a 50% hematoma rate, for which they advocate for the use of drains in the postoperative period [40]. Yap recommends the use of this flap for proximal penile shaft defects or in a staged procedure for distal defects [10].

The two-stage scrotal flap, also known as scrotal embedment or Cecil-type penile scrotal implantation, is a two-stage procedure. First, the penis is buried in the scrotum followed by resurfacing 2–4 month later. The disadvantages of the method are the awkward positioning of the penis (patients must sit on the toilet seat when urinating), the higher risk of urethral injury, and the need for two different surgeries. The advantages include lower wound dehiscence, no longitudinal scars on the dorsal aspect of the penis (meaning improved aesthetics), and lower venous congestion rates [13,15,22,39]. Nevertheless, Bajory et al. reported a 25% marginal necrosis rate after the second procedure [39].

Asanad reported a case of penile lipogranuloma with scrotal involvement, where he used a unilateral scrotal flap of the unaffected part to cover the penile shaft defect. This method could be very useful where scrotal involvement is partial and apparently healthy scrotal skin still exist. However, long-term follow-up is required, as residual foreign substance in the scrotal skin may determine the recurrence of lipogranuloma in the reconstructed area [36].

A two-stage surgical technique was employed in 39 (10.6%) patients, while the rest (n = 329) received a one-stage approach. Length of hospital stay (LoS) was reported in 16/32 papers, and data are summarized in Table 4 alongside postoperative complications and mean follow-up period.

Patient satisfaction was high in all studies. Evaluation of outcome was usually performed by applying subjective patient questionnaires: a 5-item questionnaire (1 = very unsatisfied, 2 = unsatisfied, 3 = indecisive, 4 = satisfied, 5 = very satisfied), visual analog scales, and binary “satisfied/not satisfied” answers. Few studies used a specific questionnaire for sexual function, such as the IIEF-5 [12,15,33,41] or the EHS [1,8]. Mendel et al. provided a comprehensive postoperative evaluation, in the authors’ opinion, including a 10-point Likert scale questionnaire evaluating the following items: skin coloration, sensitivity, elasticity and thickness, penile size and hairiness, scrotal volume, erection quality, penetration ability, pain, sexual satisfaction, body image, masculinity, self-esteem, and global satisfaction [1]. They also used the Patient and Observer Scar Assessment Scale (POSAS) and the Erection Hardness Score (EHS) for evaluation.

There were no studies classified as having a low risk of bias based on the Risk Of Bias In Non-randomized Studies-of Interventions (ROBINS-I) assessment. Half of the studies showed moderate and the other half high risk of bias. The risk assessment is provided in the Supplementary File S1.

## 4. Discussion

The normal appearance of the genital area is very important for psychological well-being and self-esteem. Even minor disturbances can lead to frustration, decreased sexuality and social relations. This review focuses on the use of scrotal flaps in penile resurfacing. Even though the pathology included in the study is diverse, all cases involved circumferential or partial defects involving skin, subcutaneous tissue, and dartos fascia, and the anatomical level of excision was above the Buck’s fascia.

The use of scrotal flaps for penile resurfacing offers some advantages over skin grafting or using other regional or free flaps. Because scrotal skin is very elastic and, therefore, stretchable and pliable, it offers a suitable option for penis shaft reconstruction, being able to accommodate an organ that does not stay the same size during the day. The vascular supply is very robust, which ensures good healing [26]. Moreover, the scrotal skin has the closest resemblance to penile skin, which makes these flaps a very good solution for achieving a good aesthetic and functional outcome, which is of the utmost importance for patient satisfaction. They also provide sensitive innervation by branches from the ilioinguinal nerve to the reconstructed area, ensuring protected sensation and increased sexual satisfaction [38].

The disadvantages relate to the donor site morbidity, one of the postoperative complications being scrotal contraction where large flaps are used or when insufficient scrotal size is present. Ventral contractile scars can tether the penis in an abnormal position, which can be bothersome for some patients, and this usually needs revision surgery [19]. The most undesirable feature of scrotal flaps is their hairy nature, which is aesthetically unpleasant but can be managed pre- or postoperatively with laser depilation [15]. The surgical technique for elevating and insetting the flaps can be challenging, but with careful planning, a good result is usually achievable. The flaps should be designed with the scrotal skin under stretch so as not to take too much skin and have redundancy in the recipient area, as well as to limit the reduction of scrotal size.

Wound dehiscence was the most frequent complication, encountered more when bilateral anterior scrotal artery flaps were used with a T-style anastomosis (39.4%), followed by V-style anastomosis (24%). Hematoma was a rare complication, due probably to the use of drains in the postoperative period. Due to the robust blood supply of these flaps, no total necrosis was reported. Scar contracture with abnormal penile tethering was highest in the dartos myocutaneous flaps subgroup (14.3%), followed by the bilateral scrotal flaps with T-anastomosis (10%) and scrotal pull-up technique (7.7%). This is usually of cosmetic concern, and revision surgery by z-plasty can be performed under local anesthesia with sedation.

Based on our review, it seems that bipedicled bilateral anterior scrotal flaps offer the best balance between function, aesthetics, and complication rates and are the most employed in high-volume centers. Two-stage flaps should be considered for patients requiring larger resurfacing areas and for patients at higher risk of wound dehiscence (i.e., smokers and patients with vascular diseases or diabetes). Scrotal pull-up and dartos musculocutaneous flaps seem to be better suited to smaller defects. Generalizations based on the data in the reviewed articles are impossible due to the inconsistency between techniques, surgical experiences, pre- and postoperative assessment, follow-up period.

There were few studies that performed a comprehensive outcome evaluation. For a better understanding of postoperative results, a thorough cosmetic, functional and psychological evaluation should be performed, using, in conjunction, the EHS, IIEF-5, the 10-point scale used by Mendel et al [1], and the POSAS. However, this may be hard to achieve, as the number of responders is usually low, and the rate of loss to follow-up is high. This may also be a consequence of the high number of sclerosing lipogranuloma patients, who are usually inmates or with people with poor socio-economic status.

Pre-existing conditions such as diabetes mellitus, peripheral vascular disease, and smoking history can significantly impact wound healing, infection rates, and overall surgical outcomes. Many of the included studies did not provide a detailed stratification based on comorbidities, which may be helpful so as to better decide between different techniques. Variations in surgeon expertise, training, and procedural familiarity may contribute to differences in complication rates and patient-reported satisfaction. High-volume centers may report more favorable outcomes due to greater experience.

### Limitations of the Study

The data obtained in this study, albeit including some larger case series, primarily consisted of individual or limited case reports. There were a limited number of patients who were reconstructed with a bilateral bipedicled scrotal flap with Z-plasty, with the apron-style scrotal flap, and with the dartos myocutaneous flap; therefore, larger patient numbers for these subgroups need to be evaluated to make recommendations about their utility.

Clinical and subjective assessment of voiding function was rarely reported in the studies and could not be evaluated, albeit it is an important aspect of functional reconstruction [35].

Few studies reported the mean length of hospitalization and operative time, which can serve as surrogates for the financial burden to the health system.

No comparison between different penile shaft-resurfacing methods was made, and statistical analysis could not be performed to generate evidence of the superiority of any one method.

The current literature lacks a standardized comparison between different scrotal flap techniques and with other reconstructive methods (i.e., skin grafts, artificial matrices, and other regional or free flaps). Moreover, the absence of low-risk studies, as evaluated with the ROBINS-I assessment tool, implies that all included studies had methodological limitations, having important confounding factors, selection bias, and outcome measurement issues, making the conclusions less reliable. This was either due to small sample sizes, lack of control groups, adjustment for confounders, or inconsistent outcome evaluation, which limited comparability across studies. The lack of a standardized approach for the pre- and postoperative evaluation and inconsistent reporting of complications, treatment end-point, definition of “healed from disease”, and recommended follow-up period preclude a statistical analysis of the studies included in the review.

## 5. Conclusions

Scrotal flaps provide good-quality tissue for penile resurfacing, having the closest resemblance to normal penis skin. Bipedicled bilateral anterior scrotal flaps appear to provide the most favorable balance between functional, aesthetic, and complication outcomes; however, for a better understanding of the outcomes of different subtypes of scrotal flaps, a thorough evaluation and report of postoperative complications are necessary, which may include rates of wound dehiscence, partial necrosis, scar contraction, and infection. LOS and revision surgery rates may also be important factors to consider, as they could help to address the question of the financial burden of the procedure. A routine follow-up period should be established to detect late complications. Patient satisfaction was subjectively reported as high across most studies, but standardized postoperative assessment protocols are necessary to provide comparable outcomes across different institutions. Erectile function may ideally be evaluated using a 10-item Likert scale and validated tools such as the IIEF-5, EHS, and POSAS. Furthermore, a comparative analysis of different scrotal flap techniques and other reconstructive methods would provide a stronger evidence base with which to guide surgical decision making. However, higher-quality studies are needed to establish long-term efficacy and complication profiles for different scrotal flap techniques.

## Figures and Tables

**Table 1:** Summarized description of the studies included in the review.

Study	Year	Design	No. Patients	Age (Mean) (Years)	Pathology	Technique	Complications	LOS (Days)	Follow-Up (Months)	Outcome Evaluation
Fakin [19]	2016	CS	43	37 ± 11.3	SL	Biped. V-Y	WD (19%) PN (9%) H (12%)	4.6 ± 1.8	10.7 ± 9.5	PRQ– OS (5-i) E, IC (y/n)
Muranyi [23]	2022	CS	49	33.7 ± 4.9	SL	Biped. V-Y	WD (7%) PN (16%) H (2%)	2.4 ± 1	20 ± 13.9	PRQ– OS (5-I) E, IC (y/n) Sens (y/n)
Boucher [26]	2021	CS	8	n/m	SL	Bilat. T	WD (25%)	n/m	n/m	E, IC (n/s) Sens (y/n) Aesthetic (n/s)
Kim [27]	2014	CS	5	n/m	SL	Bilat. linear	WD (20%)	4.5 ± 3.1	12	OS (n/s) E, IC (n/s) Voiding (n/s)
Le Guo [15]	2017	CS	17	35 ± 12.5	BC	2-S intrascrotal	WD (18%)	7	30 ± 12.5	PRQ– OS (5-I) Sens Voiding Aesthetic (n/s) IIEF-15
Shin [28]	2015	CR	1	48	SL	Biped. V-y	-	14	n/m	OS (n/s) E (n/s) Voiding (n/s)
Sukop [29]	2013	CR	1	36	SL	2-S intrascrotal	-	5	15	IC (y/n)
Shin [30]	2013	CS	20	43.7 ± 9.6	SL	Bilat. T	WD (100%) Scar C (10%)	n/m	n/m	E, IC (n/s) Sens (n/s)
Shin [30]	2013	CS	14	51.1 ± 11.3	SL	Bilat. V-Y	WD (21%) Scar C (7%)	n/m	n/m	E, IC (n/s) Sens (n/s)
Chu [31]	2012	CR	1	40	E/O	Apron flap	-	n/m	4	E (y/n) Sens (y/n) Aesthetic (n/s)
Shamsodini [32]	2011	CS	4	40 ± 6.5	SL	2-S intrascrtoal	WD (25%)	n/m	n/m	OS (y/n) E (y/n) Aesthetic (n/s)
Nyirady [22]	2008	CS	4	31.5 ± 5.8	SL	2-S intrascrotal	WD (50%)	n/m	24	IC (n/s) Aesthetic (n/s)
Nyirady [22]	2008	CS	12	31.5 ± 5.8	SL	Bilat. T	WD (25%)	n/m	24	IC (n/s) Aesthetic (n/s)
Gao [9]	2019	CR	1	31	C	Biped. Z	-	n/m	120	IC (y/n) Aesthetic (n/s)
Yap [10]	1993	CR	1	33	C	Dartos	Scar C (100%)	n/m	12	E, IC (y/n) Sens (y/n) Aesthetic (n/s)
Palinrungi [33]	2024	CS	32	38.8	C	Biped. V-Y	Scar C (3%)	2.2	3.4	OS (y/n) EHS
Salauddin [34]	2019	CR	1	47	SL	2-S intrascrotal	WD (100%) Scar C (100%)	n/m	n/m	OS (n/s) IIEF-5
Salauddin [34]	2019	CS	4	28.3	SL	Bilat. V-Y	WD (75%)	n/m	n/m	OS (n/s) IIEF-5
Dunev [35]	2019	CR	1	18	SL	Bilat. V-Y	-	7	84	E, IC (n/s) Sens (y/n) Aesthetic (n/s)
Manjit [8]	2015	CR	1	32	SL	Bilat. T	-	3	3	IC (y/n)
De Siati [36]	2013	CR	1	27	SL	Bilat. T	-	2	12	E, IC (n/s) Voiding (n/s) Aesthetic (n/s)
Huang [11]	2021	CR	1	44	PNeo	Scrotal Pull-up	WD (100%) Scar C (100%)	3	6	E (n/s)
Asanad [37]	2018	CR	1	56	SL	Unilateral scrotal flap	n/m	n/m	n/m	OS (n/s) E (y/n) Aesthetic (n/s)
Han [17]	2015	CS	12	19.9 ± 12.6	CoP	Scrotal Pull-up	-	n/m	27.4 ± 12.6	PRQ– OS (5-I) Voiding (n/s) Aesthetic (n/s)
Mahadewa [6]	2023	CR	1	19	SL	Biped. V-Y	-	2	1.5	OS (n/s) E (n/s) Aesthetic (n/s)
Zucchi [13]	2010	CS	10	10 ± 35.5	CoP	2-S intrascrtoal	H (10%) ED (20%)	n/m	20	OS (VAS-100) E, IC (n/s)
Son [38]	2023	CR	1	41	SL	Bilat. T	Scar C (100%)	n/m	3	OS (VAS–10) E, IC (y/n) Sens (y/n)
Jeong [39]	1996	CS	17	34.4	SL	Bilat. T	WD (23%) H (6%) Scar C (12%) ED (12%)	6 ± 1	14.8	IC (n/s) Sens (n/s)
Bajory [40]	2013	CS	20	n/m	SL	2-S intrascrotal	-	8.7 ± 5.4	n/m	OS (n/s) IC (n/s) Aesthetic (n/s)
Bajory [40]	2013	CS	12	n/m	SL	Bilat. T	WD (25%) PN (25%) Uretr. Fist. (5%)	8.7 ± 5.4	n/m	OS (n/s) IC (n/s) Aesthetic (n/s)
Xie [12]	2024	CS	5	57.4 ± 7.8	Ring Inc	Bilat. T	Uretr. Fist. (20%)	n/m	6	IIEF-5 Aesthetic (n/s)
Tiwari [41]	1991	CS	6	27.8 ± 7.3	BC	Dartos	PN (17%) H (50%)	n/m	6	Sens (y/n)
Zhao [14]	2009	CS	12	26.4	Multiple	Biped. V-Y	PN (6%) ED (11%)	n/m	27	OS (n/s) E, IC (n/s) Sens (n/s) Voiding (n/s)
Zhao [14]	2009	CS	6	26.4	Multiple	Bilat. V-Y	-	n/m	27	OS (n/s) E, IC (n/s) Sens (n/s) Voiding (n/s)
Westerman [16]	2015	CS	15	51	CoP	Bilat. linear	-	1	12	PRQ– E (n/s)
Napolitano [5]	2023	CR	1	23	SL	Biped. V-Y	-	7	6	OS (n/s) Voiding (n/s) Aesthetic (n/s)
Yao [41]	2022	CS	5	35 ± 10.2	Trauma	Bilat. linear	PN (28.6%)	n/m	n/m	IIEF-5
Mendel [1]	2023	CS	22	48.9 ± 19.3	Multiple	Bilat. T	WD (32%) H (5%) Scar C (27%)	4.6 ± 3	43.7 ± 30.4	PRQ–(10-I Aesthetic, Sens, E, IC, Psy, OS) EHS POSAS

Design: CR case report, CS case series. Pathology: BC burn contracture, C circumcision, CoP concealed penis, E/O epididimitis/orchitis, SL sclerosing lipogranuloma, PNeo penile neoplasia, Ring Inc ring incarceration. Technique: 2-S intrascrotal two-stage intrascrotal, Biped. V-Y bipedicled scrotal flaps with V-Y closure, Biped. Z bipedicled scrotal flap with Z-plasty closure, Bilat. linear bilateral scrotal flap with linear closure, Bilat. T bilateral scrotal flaps with T closure, Bilat. V-Y bilateral scrotal flaps with V-Y closure, Dartos island dartos myocutaneous flap. Complications: ED erectile disfunction, H hematoma, PN partial necrosis, Scar C scar contracture, Uretr. Fist urethral fistula, WD wound dehiscence; LOS length of hospital stay. Outcome evaluation: E erection, EHS erection hardness score, IC intercourse, IIEF-5 International Index of Erectile Function, n/s not stated, OS overall satisfaction, PRQ patient-reported questionnaire, Sens. Sensibility, y/n yes/no.

**Table 2:** Number of patients for each pathology described.

Disease	No. Patients (%)
Sclerosing lipogranuloma	261 (71%)
Concealed penis	43 (11.7%)
Burn contracture	23 (6.2%)
Neoplasia	14 (3.8%)
Animal bite	9 (2.4%)
Circumcision	8 (2.1%)
Trauma	5 (1.4%)
Ring incarceration	5 (1.4%)

**Table 3:** Presenting complications in the sclerosing lipogranuloma group.

Complication	No. (%)
Infection	28 (24.6)
Painful erection/erectile disfunction	62 (54.4)
Constant discomfort/pain	54 (47.4)
Skin ulcer	49 (33.6)
Phimosis	39 (26.7)
Persistent inflammation	75 (51.4)

**Table 4:** Complications and postoperative data regarding different types of scrotal flap reconstruction for penile shaft defects.

	Bilateral Anterior Scrotal Flaps (T-Style Anastomosis) (n = 99)	Bilateral Anterior Scrotal Flaps (V-Y Anastomosis) (n = 25)	Bilateral Anterior Scrotal (Linear Ventral Scar) (n = 25)	Bilateral Bipedicled Scrotal Flap (V-Y) (n = 139)	Bilateral Bipedicled Scrotal Flap (z Plasty) (n = 1)	Scrotal Pull-Up (n = 13)	Apron-Style Scrotal Flap (n = 1)	Dartos Myocutaneous Flap (n = 7)	Two-Stage Intrascrotal (n = 57)
Mean LoS (days)	5.9	n/m	2	3.2	n/m	3	n/m	n/m	7.8
Wound dehiscence	39.4%	24%	4%	8%	0%	7.7%	0%	0%	12.3%
Total necrosis	0%	0%	0%	0%	0%	0%	0%	0%	0%
Partial necrosis	3%	0%	4%	9%	0%	%	0%	14.3%	0%
Hematoma	2%	0%	0%	4.3%	0%	0%	0%	42.9%	1.7%
Scar contracture	10%	4%	0%	2.8%	0%	7.7%	0%	14.3%	1.7%
Erectile disfunction	2%	0%	0%	6.5%	0%	0%	0%	0%	7%
Urethral fistula	2%	0%	0%	0%	0%	0%	0%	0%	0%
Revision surgery	13%	4%	8%	3.9%	0%	0%	100%	0%	100%
Follow-up (months)	15.6	27	12	13.6	120	25.8	4	6.9	25.7

## Data Availability

The raw data obtained during the review are available from the corresponding author upon reasonable request.

## Supplementary Materials

The following supporting information can be downloaded at: https://www.mdpi.com/article/10.3390/medicina61061052/s1, File S1: Bias assessment table using ROBINS-I.
