# Supplementary material for: Scrotal Flaps for Penile Skin Reconstruction: A Systematic Review
Source: Medicina (Kaunas). 2025 Jun 6;61(6):1052. doi: 10.3390/medicina61061052 (PMC12195437; doi:10.3390/medicina61061052)
Supplement: Supplementary file 1 [file medicina-61-01052-s001.zip › medicina-3673154-supplementary.pdf]

Bias assessment table using ROBINS-I

Study	Study Type	Number of Patients	Selection Bias	Confounding Bias	Measurement Bias	Overall Risk of Bias
**Fakin et al. (2016)**	Case Series	43	Moderate	Moderate	Low	Moderate
**Muranyi et al. (2022)**	Case Series	49	Moderate	High	Moderate	High
**Boucher et al. (2021)**	Case Series	8	Moderate	Moderate	Low	Moderate
**Kim et al. (2014)**	Case Series	5	Moderate	Moderate	Low	Moderate
**Guo et al. (2017)**	Case Series	17	Moderate	Moderate	Low	Moderate
**Shin et al. (2015)**	Case Report	1	High	High	Moderate	High
**Sukop et al. (2013)**	Case Report	1	High	High	Moderate	High
**Shin et al. (2013)**	Case Series	34	Moderate	Moderate	Low	Moderate
**Chu et al. (2012)**	Case Report	1	High	High	Moderate	High
**Shamsodini et al. (2011)**	Case Series	4	Moderate	High	Moderate	High
**Nyirády et al. (2008)**	Case Series	16	Moderate	Moderate	Low	Moderate
**Gao et al. (2019)**	Case Report	1	High	High	Moderate	High
**Yap et al. (1993)**	Case Report	1	High	High	Moderate	High
**Palinrungi et al. (2024)**	Case Series	32	Moderate	Moderate	Low	Moderate
**Salauddin et al. (2019)**	Case Series	5	Moderate	High	Moderate	High
**Manjit et al. (2015)**	Case Report	1	High	High	Moderate	High
**De Siati et al. (2013)**	Case Report	1	High	High	Moderate	High
**Huang et al. (2021)**	Case Report	1	High	High	Moderate	High
**Asanad et al. (2018)**	Case Report	1	High	High	Moderate	High
**Han et al. (2015)**	Case Series	12	Low	Moderate	Low	Moderate
**Mahadewa et al. (2023)**	Case Report	1	High	High	Moderate	High
**Zucchi et al. (2010)**	Case Series	10	Moderate	High	Moderate	High
**Son et al. (2023)**	Case Report	1	High	High	Moderate	High
**Jeong et al. (1996)**	Case Series	17	Moderate	Moderate	Low	Moderate
**Bajory et al. (2013)**	Case Series	32	Moderate	Moderate	Low	Moderate
**Xie et al. (2024)**	Case Series	5	Moderate	Moderate	Low	Moderate
**Tiwari et al. (1991)**	Case Series	6	Moderate	Moderate	Low	Moderate
**Zhao et al. (2009)**	Case Series	18	Moderate	Moderate	Low	Moderate
**Westerman et al. (2015)**	Case Series	15	Moderate	Moderate	Low	Moderate
**Napolitano et al. (2023)**	Case Report	1	High	High	Moderate	High
**Yao et al. (2022)**	Case Series	5	Moderate	Moderate	Low	Moderate
**Mendel et al. (2023)**	Case Series	22	Moderate	Moderate	Low	Moderate
